# Supplementary material for: Design, Synthesis and Evaluation of New Indolylpyrimidylpiperazines for Gastrointestinal Cancer Therapy
Source: Molecules. 2019 Oct 11;24(20):3661. doi: 10.3390/molecules24203661 (PMC6832257; doi:10.3390/molecules24203661)
Supplement: Supplementary file 1 [file molecules-24-03661-s001.pdf]

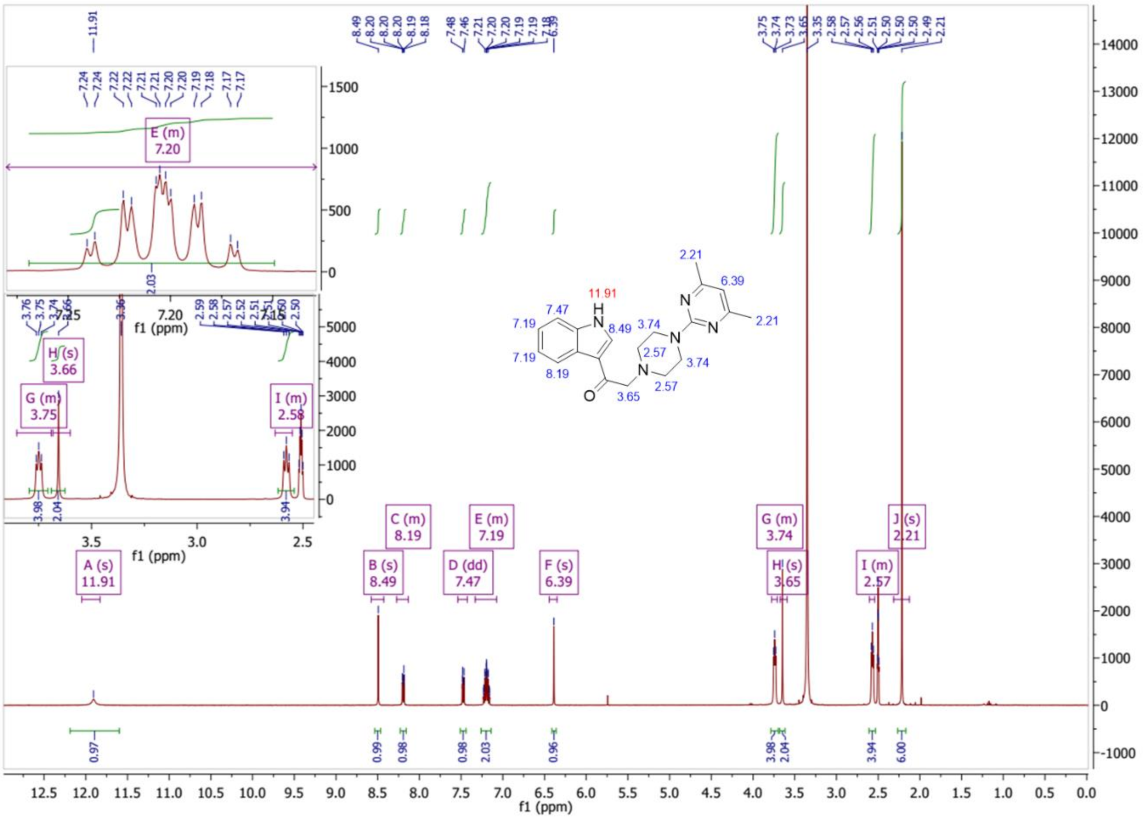

Figure S1: ^{1}H NMR of Compound 3.

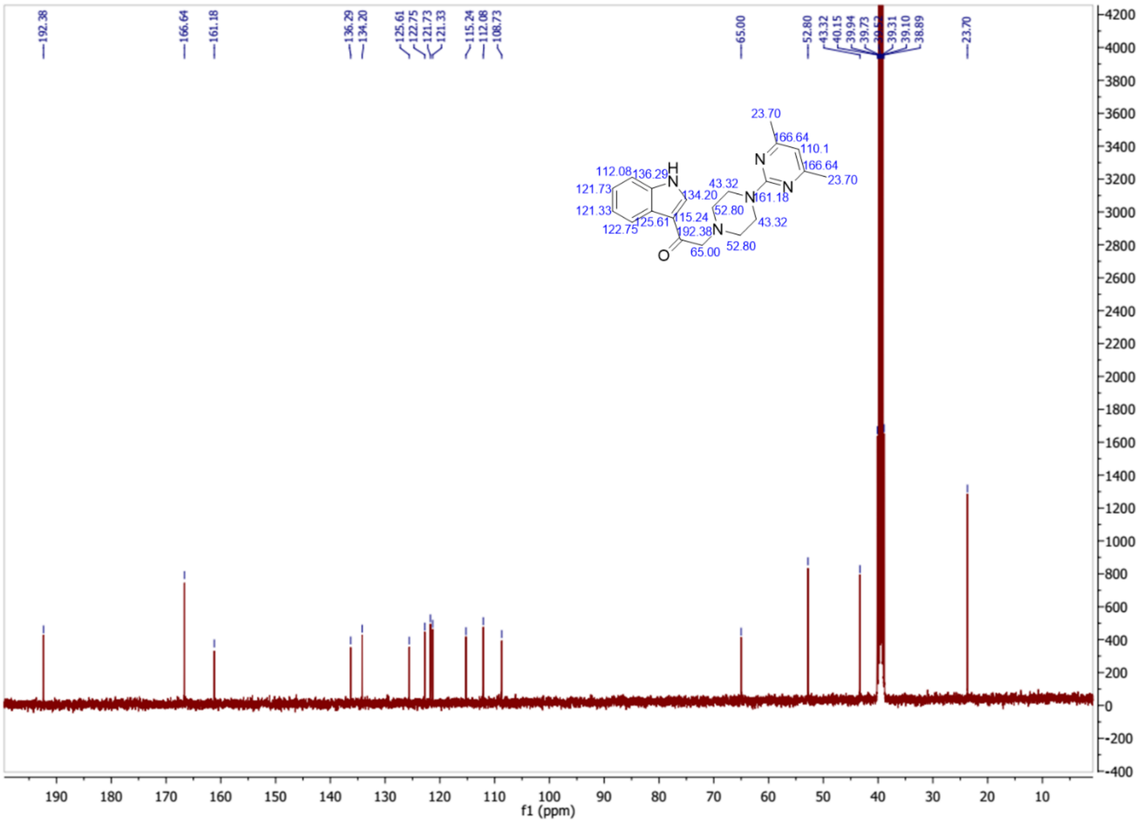

Figure S2: ^{13}C NMR of Compound 3.

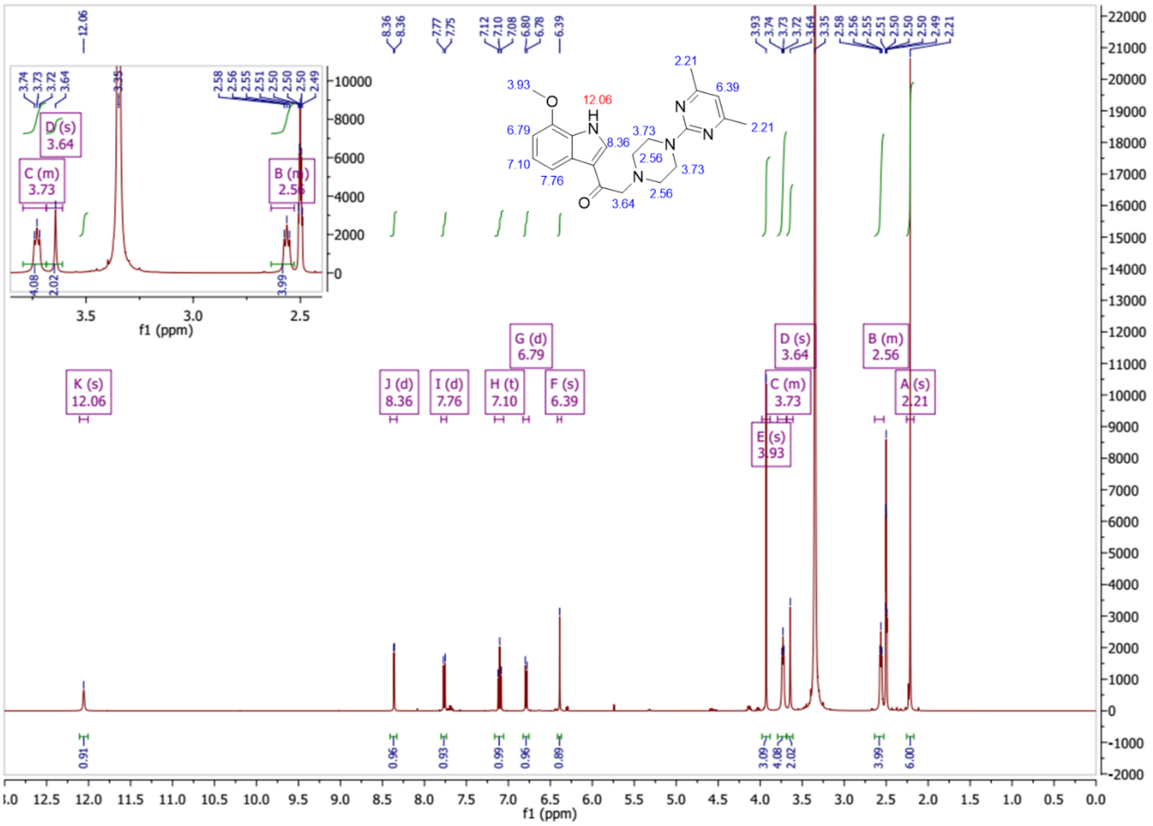

Figure S3: ^{1}H NMR of Compound 4.

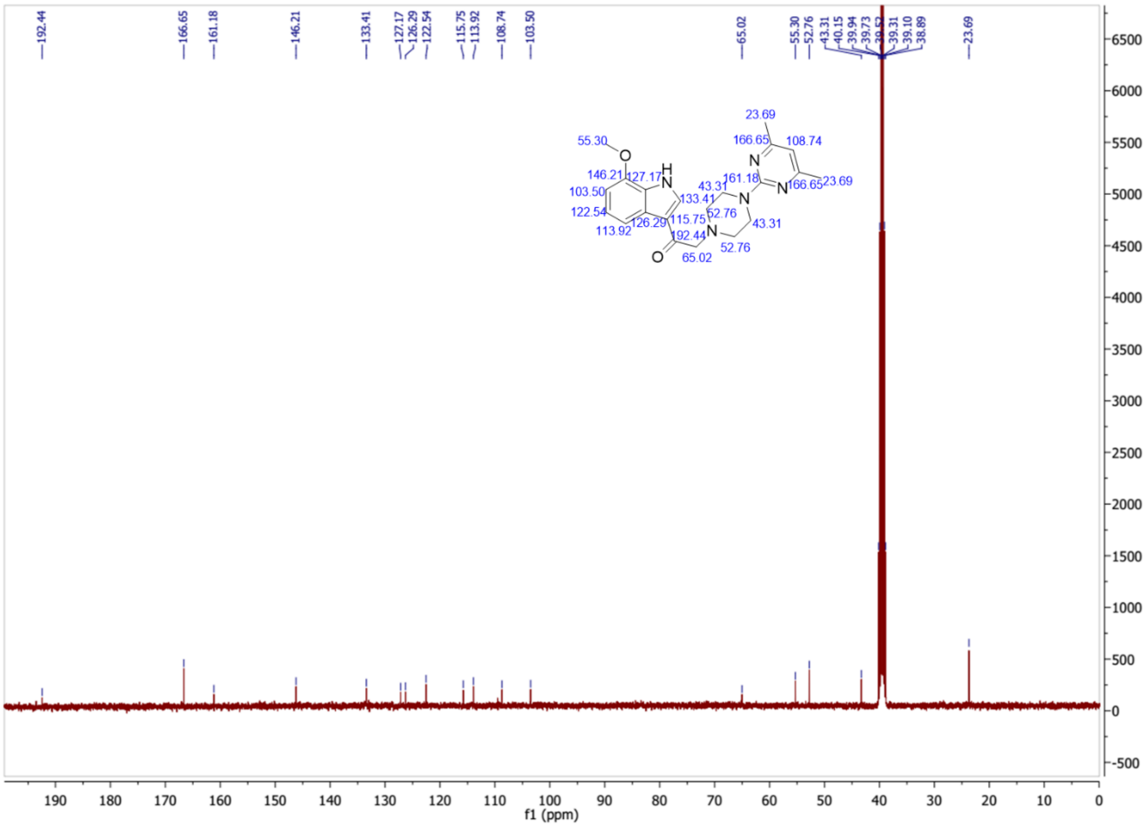

Figure S4: ^{13}C NMR of Compound 4.

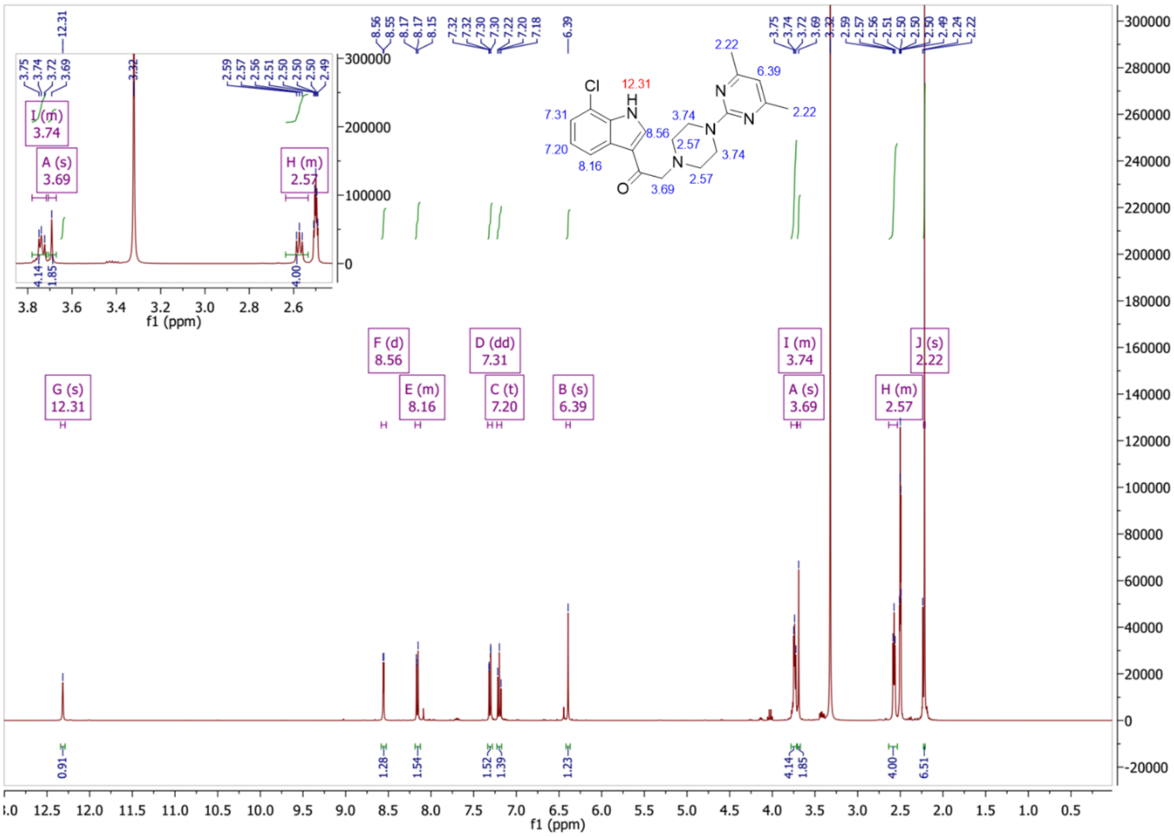

Figure S5: 1H NMR of Compound 5.

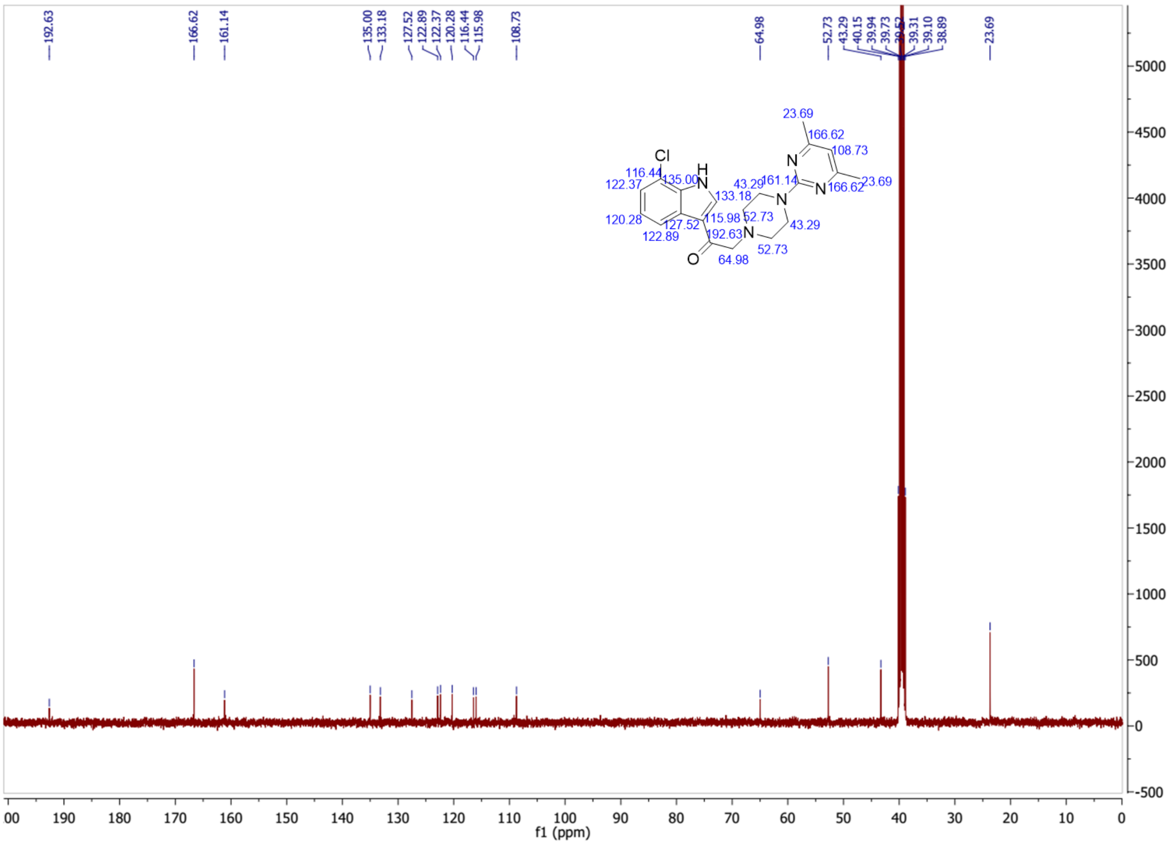

Figure S6: 13C NMR of Compound 5.

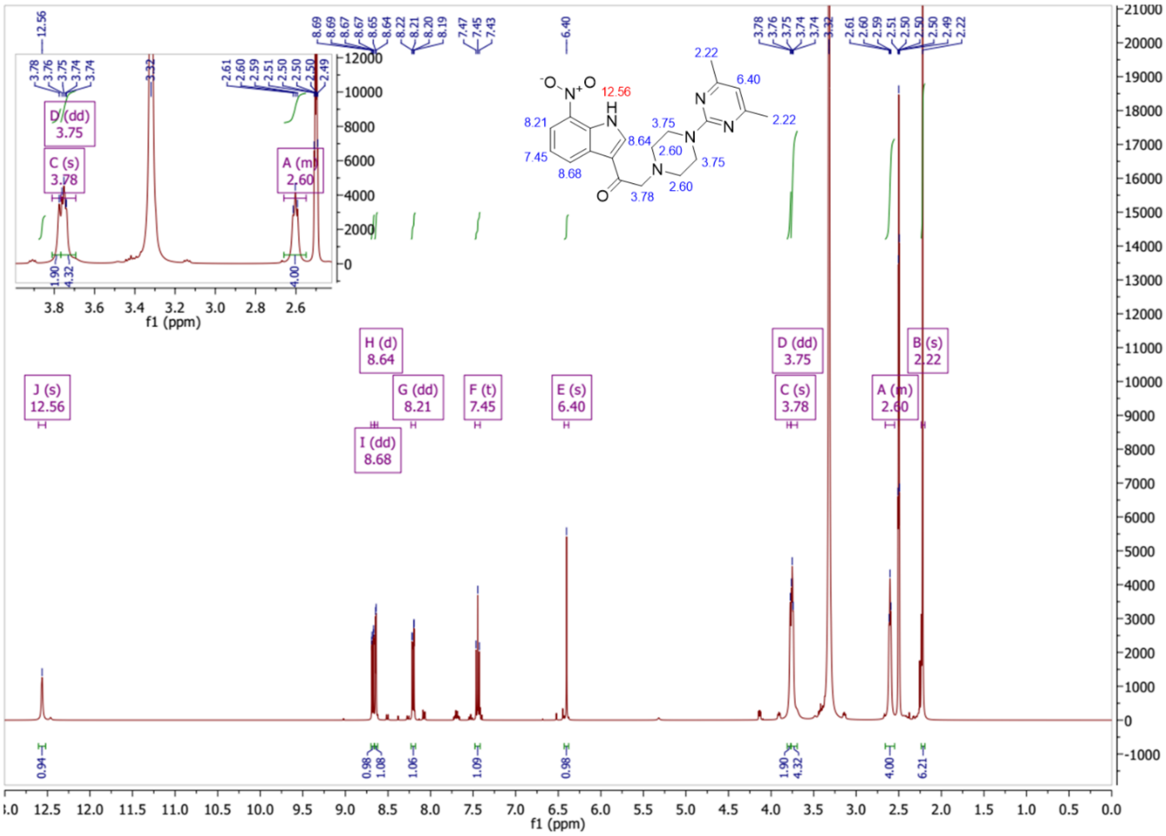

Figure S7: ^{1}H NMR of Compound 6.

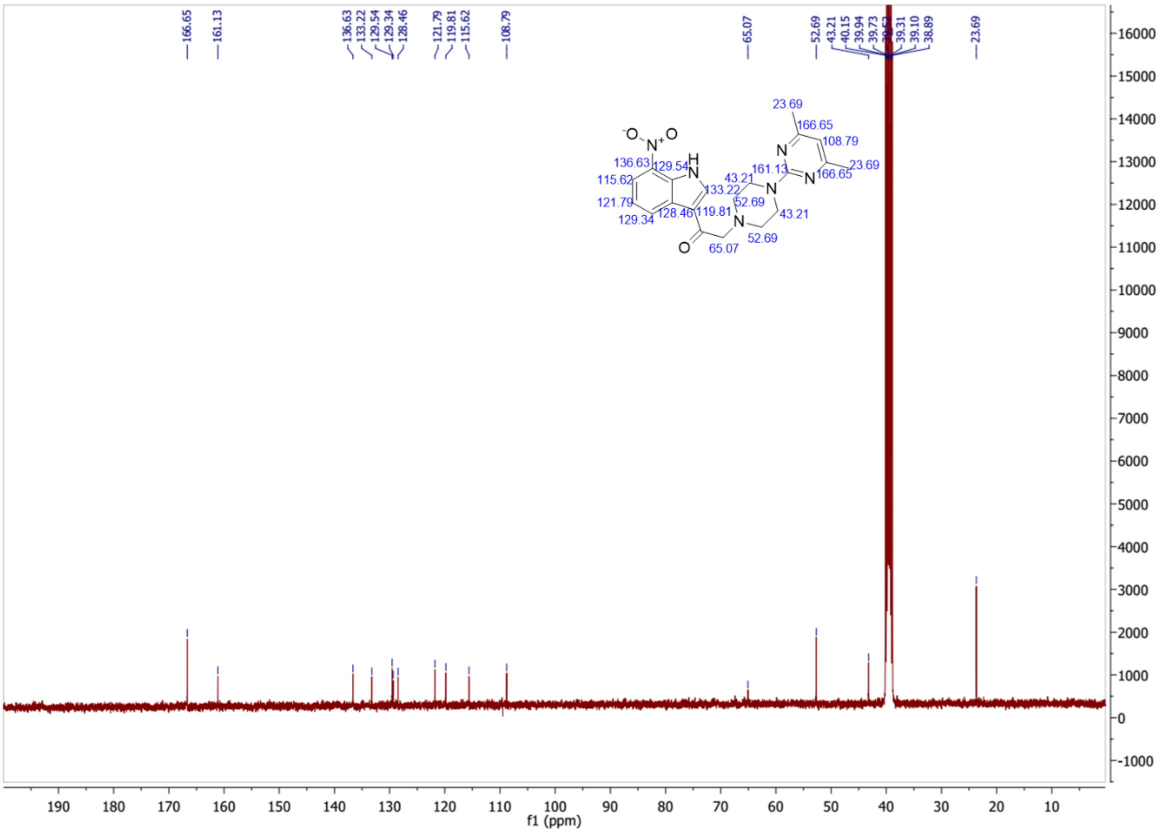

Figure S8: ^{13}C NMR of Compound 6.

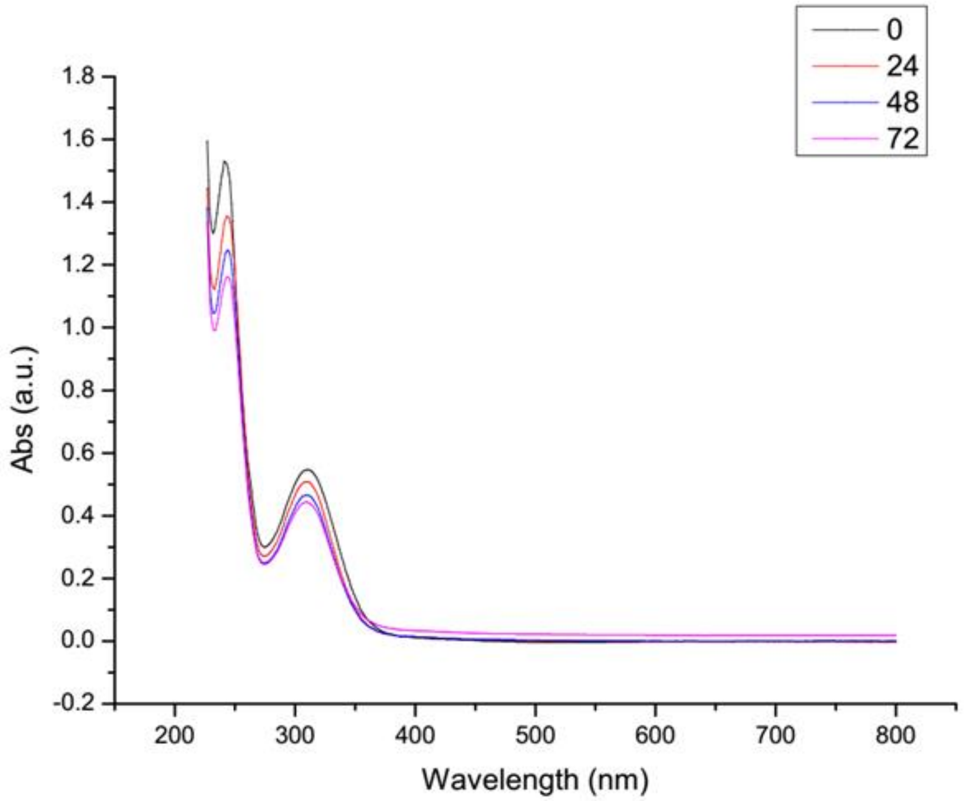

Figure S9: UV-stability of Compound 4 in water over 72h.

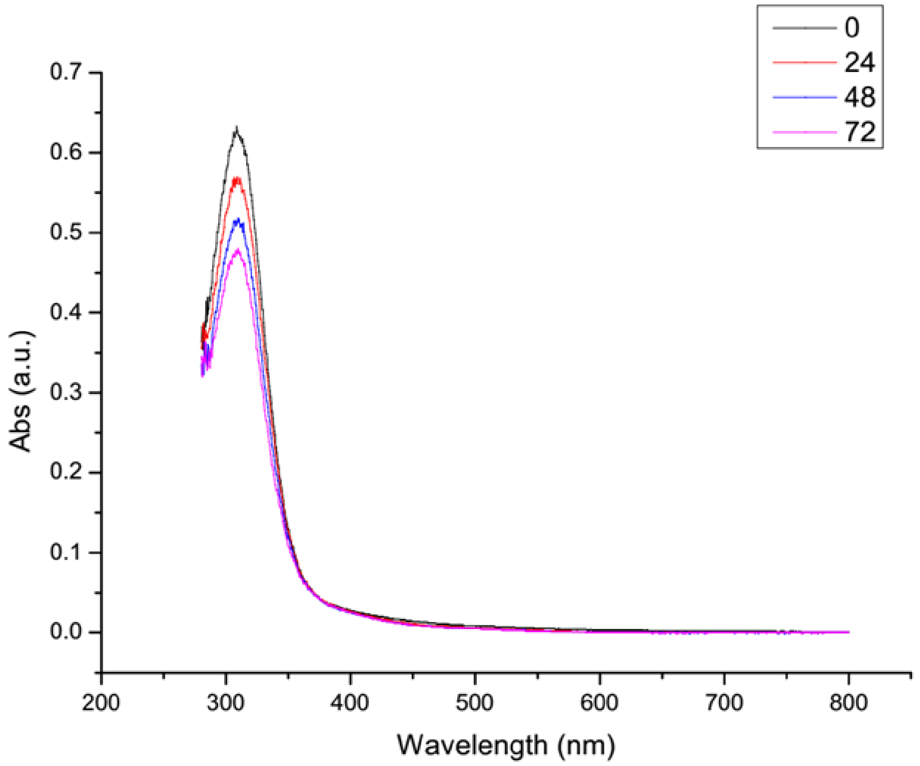

Figure S10: UV-stability of Compound 4 in DMEM over 72h.

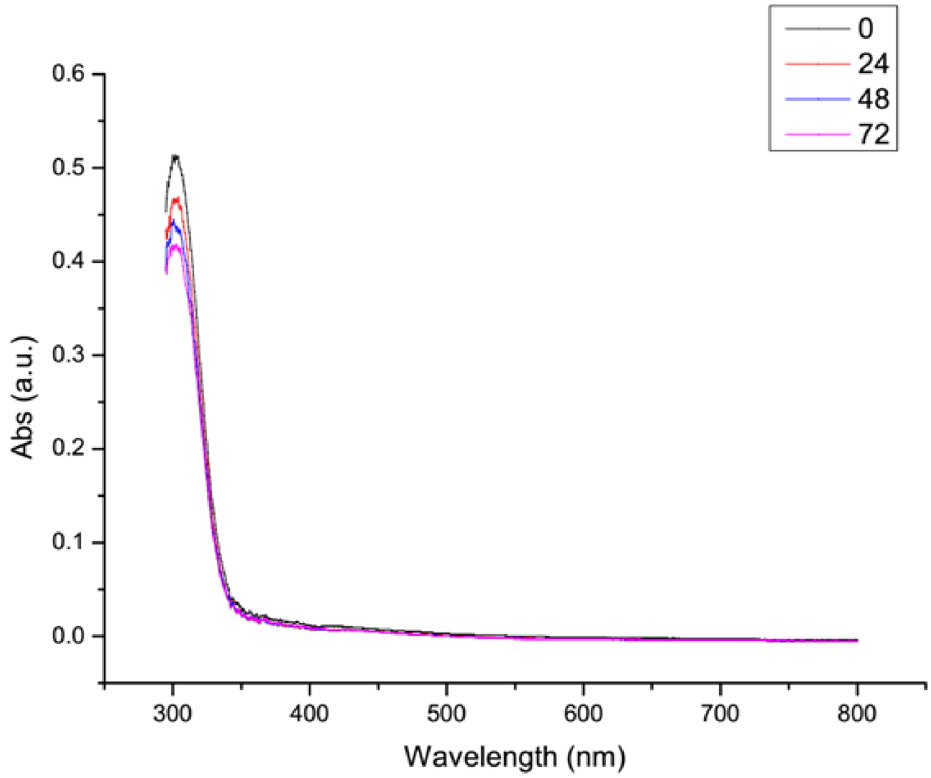

Figure S11: UV-stability of Compound 4 in RPMI-1640 over 72h.

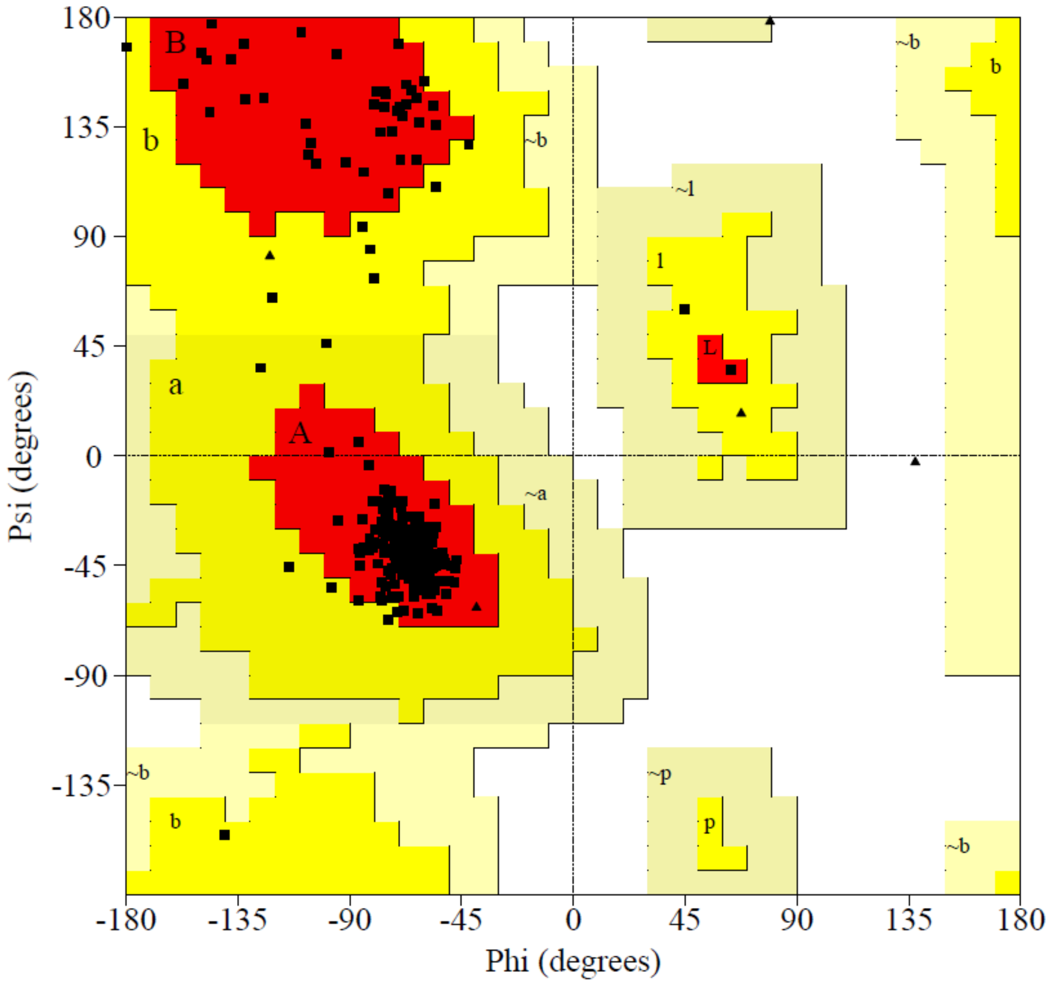

Plot statistics

Residues in most favoured regions [A,B,L]	268	94.7%
Residues in additional allowed regions [a,b,l,p]	15	5.3%
Residues in generously allowed regions [~a,~b,~l,~p]	0	0.0%
Residues in disallowed regions	0	0.0%
	----	------
Number of non-glycine and non-proline residues	283	100.0%
Number of end-residues (excl. Gly and Pro)	2	
Number of glycine residues (shown as triangles)	11	
Number of proline residues	8	

Total number of residues	304	

Based on an analysis of 118 structures of resolution of at least 2.0 Angstroms and R-factor no greater than 20%, a good quality model would be expected to have over 90% in the most favoured regions.

Figure S12: Ramachandran plot for hA_3AR homology model (PROCHECK analysis).
